# Diaqua­bis­{5-(pyridin-2-yl-κ*N*)-3-[4-(pyri­din-4-yl)phenyl]-1*H*-1,2,4-triazol-1-ido-κ*N* ^1^}zinc

**DOI:** 10.1107/S1600536813005916

**Published:** 2013-03-16

**Authors:** Bin Li

**Affiliations:** a Advanced Material Institute of Research, Department of Chemistry and Chemical Engineering, Qilu Normal University, Zhangqiu 250200, People’s Republic of China

## Abstract

The asymmetric unit of the title compound, [Zn(C_18_H_12_N_5_)_2_(H_2_O)_2_], consists a Zn^II^ ion, located on an inversion center, a deprotonated 5-pyridin-2-yl-3-[4-(pyridin-4-yl)phen­yl]-1*H*-1,2,4-triazol-1-ido ligand and a water mol­ecule. The whole mol­ecule is generated by inversion symmetry. The Zn^II^ ion has a distorted octa­hedral coordination geometry, defined by four N atoms from the two deprotonated organic ligands and two water O atoms. In the crystal, O—H⋯N hydrogen bonds link the mol­ecules, forming a three-dimensional network.

## Related literature

For background to coordination complexes, see: Zhang *et al.* (2012*a* ▶,*b* ▶); Fan *et al.* (2013 ▶).
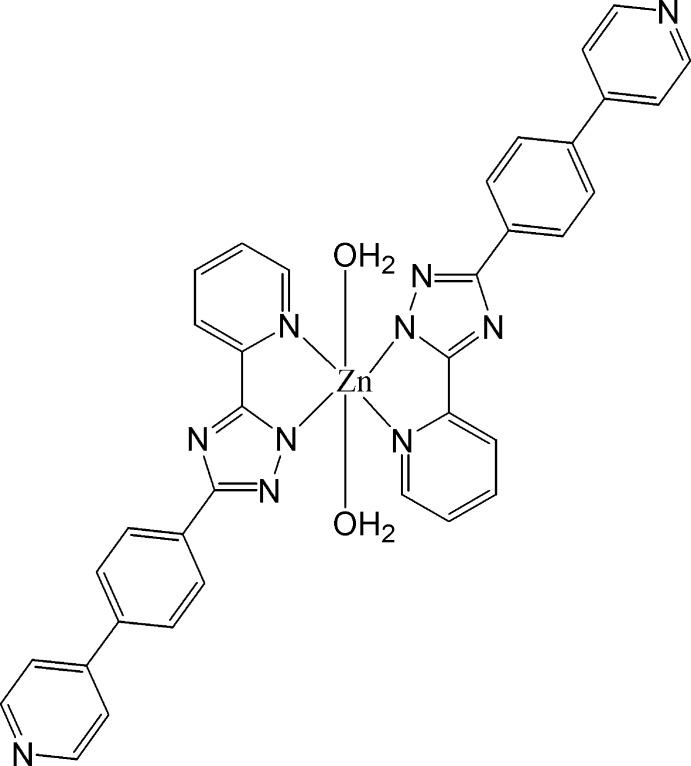


## Experimental

### 

#### Crystal data

[Zn(C_18_H_12_N_5_)_2_(H_2_O)_2_]
*M* *_r_* = 698.05
Monoclinic, 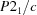

*a* = 13.214 (5) Å
*b* = 12.049 (5) Å
*c* = 9.825 (4) Å
β = 100.709 (3)°
*V* = 1537.0 (10) Å^3^
*Z* = 2
Mo *K*α radiation
μ = 0.85 mm^−1^
*T* = 296 K
0.12 × 0.10 × 0.08 mm

#### Data collection

Bruker APEXII CCD area-detector diffractometer
Absorption correction: multi-scan (*SADABS*; Bruker, 2001 ▶) *T*_min_ = 0.905, *T*_max_ = 0.935
7962 measured reflections
2718 independent reflections
1731 reflections with *I* > 2σ(*I*)
*R*_int_ = 0.070

#### Refinement

*R*[*F* ^2^ > 2σ(*F* ^2^)] = 0.057
*wR*(*F* ^2^) = 0.151
*S* = 1.00
2718 reflections
229 parameters
H atoms treated by a mixture of independent and constrained refinement
Δρ_max_ = 0.65 e Å^−3^
Δρ_min_ = −0.32 e Å^−3^

### 

Data collection: *APEX2* (Bruker, 2004 ▶); cell refinement: *SAINT-Plus* (Bruker, 2001 ▶); data reduction: *SAINT-Plus*; program(s) used to solve structure: *SHELXS97* (Sheldrick, 2008 ▶); program(s) used to refine structure: *SHELXL97* (Sheldrick, 2008 ▶); molecular graphics: *SHELXTL* (Sheldrick, 2008 ▶) and *Mercury* (Macrae *et al.*, 2008 ▶); software used to prepare material for publication: *SHELXTL*.

## Supplementary Material

Crystal structure: contains datablock(s) global, I. DOI: 10.1107/S1600536813005916/su2568sup1.cif

Structure factors: contains datablock(s) I. DOI: 10.1107/S1600536813005916/su2568Isup2.hkl

Additional supplementary materials:  crystallographic information; 3D view; checkCIF report

## Figures and Tables

**Table 1:** Hydrogen-bond geometry (Å, °)

*D*—H⋯*A*	*D*—H	H⋯*A*	*D*⋯*A*	*D*—H⋯*A*
O1—H1*W*⋯N3^i^	0.75 (6)	2.07 (6)	2.812 (5)	169 (6)
O1—H2*W*⋯N5^ii^	0.85 (6)	2.38 (6)	3.165 (7)	155 (6)

Symmetry codes: (i) 
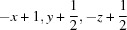
; (ii) 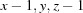.

**Fig. 1.**
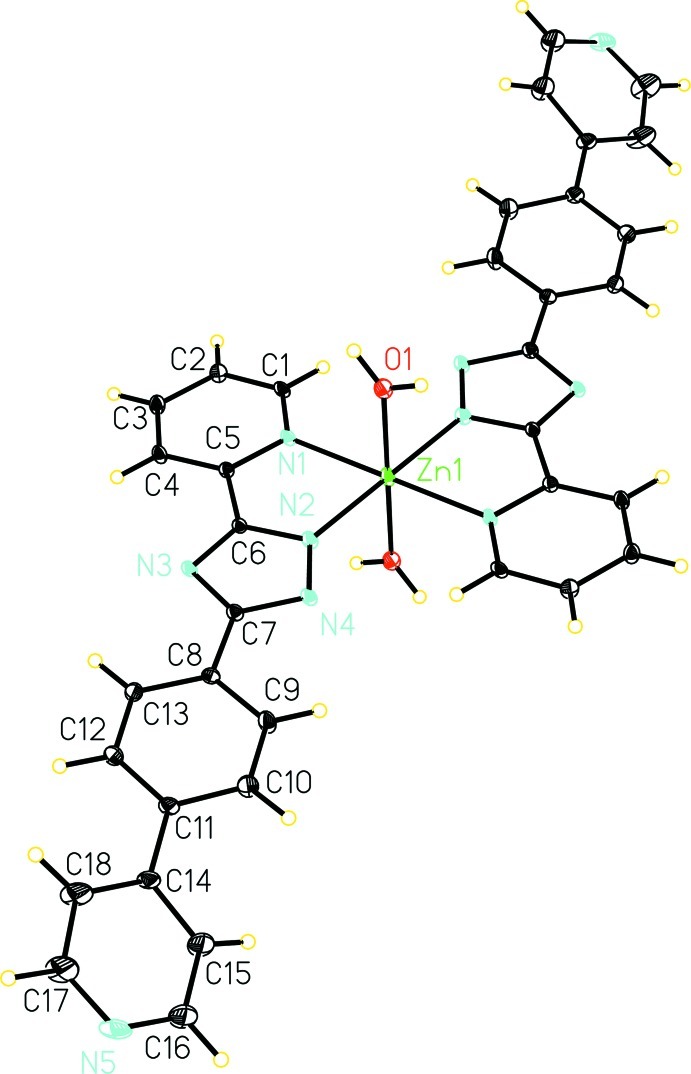
The molecular structure of the title compound, with the atom-labelling. Displacement ellipsoids are drawn at the 30% probability level.

**Fig. 2.**
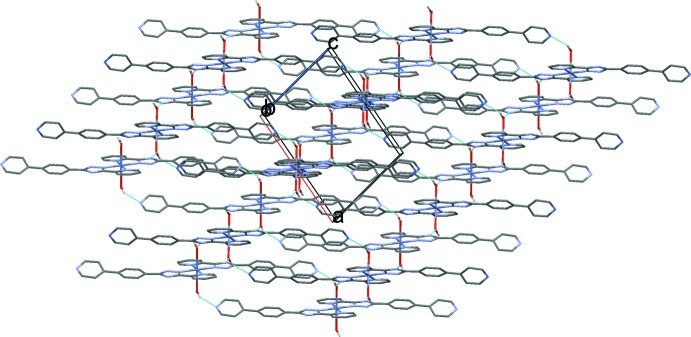
The crystal packing of the title compound viewed along the b axis. Hydrogen bonds are shown as dashed cyan lines (see Table 1 for details; C-bound H atoms have been omitted for clarity).

## supplementary crystallographic information

### Comment

The design and synthesis of coordination complexes has attracted upsurging research interest not only because of their appealing structural and topological novelty but also owing to their potential applications in gas storage, microelectronics, ion exchange, chemical separations, nonlinear optics and heterogeneous catalysis. Here, we report on the complex formed by a solvothermal reaction of 2-(3-(4-(pyridin-4- yl)phenyl)-1H-1,2,4-triazol-5-yl)pyridine with zinc(II) acetate.

The title compound, Fig. 1, possesses inversion symmetry, and consists of a zinc atom (located on the inversion center) coordinated to two symmetry related deprotonated 2-(3-(4-(pyridin-4- yl)phenyl)-1H-1,2,4-triazol-5-yl)pyridine ligands and two water molecules. The zinc atom, Zn1, has a distorted ZnN4O2 octahedral coordination geometry; completed by four N atoms of the ligand and two O atoms from the two water molecules. The Zn1—O1 distance is 2.301 (4) Å, and the Zn—N distances varying from 2.048 (3) - 2.134 (3) Å.

In the crystal, O-H···N hydrogen bonds link the molecules forming a three-dimensional network (Fig. 2 and Table 1).

### Experimental

A mixture of 2-(3-(4-(pyridin-4- yl)phenyl)-1H-1,2,4-triazol-5-yl)pyridine (0.20 mmol, 0.060 g), zinc acetate dihydrate (0.20 mmol, 0.044 g) and NaOH (0.20 mmol, 0.008 g) in 12 mL H_2_O was placed in a Teflon-lined stainless steel vessel and heated to 443 K for 3 days, followed by slow cooling (a descent rate of 10 K/h) to room temperature. Colourless block-like crystals suitable for X-ray diffraction analysis were obtained. Anal. Calc. for C_36_H_28_ZnN_10_O_2_: C 61.94, H 4.04, N 20.06%; Found: C 61.89, H 4.01, N 19.97%.

### Refinement

The C-bound H atoms were included in calculated positions refined using a riding model: C—H = 0.93 Å with *U*_iso_ = 1.2*U*_eq_(C). The water H atoms were located in difference electron density maps and refined with distance restraints: O—H = 0.83 (2) Å and *U*_iso_(H) fixed at 0.80 Å^2^.

### Crystal data

**Table:**

[Zn(C_18_H_12_N_5_)_2_(H_2_O)_2_]	*F*(000) = 720
*M**_r_* = 698.05	*D*_x_ = 1.508 Mg m^−^^3^
Monoclinic, *P*2_1_/*c*	Mo *K*α radiation, λ = 0.71073 Å
Hall symbol: -P 2ybc	Cell parameters from 985 reflections
*a* = 13.214 (5) Å	θ = 2.3–20.2°
*b* = 12.049 (5) Å	µ = 0.85 mm^−^^1^
*c* = 9.825 (4) Å	*T* = 296 K
β = 100.709 (3)°	Block, colorless
*V* = 1537.0 (10) Å^3^	0.12 × 0.10 × 0.08 mm
*Z* = 2	

### Data collection

**Table:**

Bruker APEXII CCD area-detector diffractometer	2718 independent reflections
Radiation source: fine-focus sealed tube	1731 reflections with *I* > 2σ(*I*)
Graphite monochromator	*R*_int_ = 0.070
phi and ω scans	θ_max_ = 25.0°, θ_min_ = 2.3°
Absorption correction: multi-scan (*SADABS*; Bruker, 2001)	*h* = −15→14
*T*_min_ = 0.905, *T*_max_ = 0.935	*k* = −14→14
7962 measured reflections	*l* = −11→9

### Refinement

**Table:**

Refinement on *F*^2^	Primary atom site location: structure-invariant direct methods
Least-squares matrix: full	Secondary atom site location: difference Fourier map
*R*[*F*^2^ > 2σ(*F*^2^)] = 0.057	Hydrogen site location: inferred from neighbouring sites
*wR*(*F*^2^) = 0.151	H atoms treated by a mixture of independent and constrained refinement
*S* = 1.00	*w* = 1/[σ^2^(*F*_o_^2^) + (0.035*P*)^2^ + 1.3183*P*] where *P* = (*F*_o_^2^ + 2*F*_c_^2^)/3
2718 reflections	(Δ/σ)_max_ = 0.001
229 parameters	Δρ_max_ = 0.65 e Å^−^^3^
0 restraints	Δρ_min_ = −0.32 e Å^−^^3^

### Special details

**Table:**

Geometry. All esds (except the esd in the dihedral angle between two l.s. planes) are estimated using the full covariance matrix. The cell esds are taken into account individually in the estimation of esds in distances, angles and torsion angles; correlations between esds in cell parameters are only used when they are defined by crystal symmetry. An approximate (isotropic) treatment of cell esds is used for estimating esds involving l.s. planes.
Refinement. Refinement of F^2^ against ALL reflections. The weighted R-factor wR and goodness of fit S are based on F^2^, conventional R-factors R are based on F, with F set to zero for negative F^2^. The threshold expression of F^2^ > 2sigma(F^2^) is used only for calculating R-factors(gt) etc. and is not relevant to the choice of reflections for refinement. R-factors based on F^2^ are statistically about twice as large as those based on F, and R- factors based on ALL data will be even larger.

### Fractional atomic coordinates and isotropic or equivalent isotropic displacement parameters (Å^2^)

**Table:**

	*x*	*y*	*z*	*U*_iso_*/*U*_eq_	
C1	0.3744 (3)	0.7926 (3)	−0.1269 (4)	0.0397 (11)	
H1	0.3415	0.8410	−0.1946	0.048*	
C2	0.3495 (4)	0.6829 (4)	−0.1378 (5)	0.0444 (12)	
H2	0.3016	0.6568	−0.2124	0.053*	
C3	0.3963 (4)	0.6118 (4)	−0.0371 (5)	0.0474 (13)	
H3	0.3800	0.5366	−0.0424	0.057*	
C4	0.4679 (4)	0.6519 (3)	0.0729 (5)	0.0412 (11)	
H4	0.5001	0.6047	0.1426	0.049*	
C5	0.4902 (3)	0.7630 (3)	0.0761 (4)	0.0317 (10)	
C6	0.5671 (3)	0.8172 (3)	0.1839 (4)	0.0308 (10)	
C7	0.6829 (3)	0.8610 (3)	0.3510 (4)	0.0360 (10)	
C8	0.7641 (3)	0.8613 (4)	0.4753 (4)	0.0373 (11)	
C9	0.8225 (4)	0.9560 (4)	0.5076 (5)	0.0619 (16)	
H9	0.8083	1.0183	0.4514	0.074*	
C10	0.9010 (4)	0.9603 (4)	0.6208 (6)	0.0636 (16)	
H10	0.9395	1.0250	0.6386	0.076*	
C11	0.9241 (4)	0.8705 (4)	0.7089 (5)	0.0459 (12)	
C12	0.8626 (4)	0.7784 (4)	0.6780 (5)	0.0540 (14)	
H12	0.8747	0.7172	0.7364	0.065*	
C13	0.7839 (4)	0.7727 (4)	0.5648 (5)	0.0468 (12)	
H13	0.7440	0.7088	0.5487	0.056*	
C14	1.0114 (4)	0.8769 (4)	0.8265 (5)	0.0523 (13)	
C15	1.0930 (5)	0.9456 (6)	0.8211 (7)	0.091 (2)	
H15	1.0946	0.9868	0.7415	0.109*	
C16	1.1734 (5)	0.9543 (6)	0.9339 (8)	0.091 (2)	
H16	1.2270	1.0026	0.9262	0.109*	
C17	1.1083 (6)	0.8258 (8)	1.0459 (7)	0.123 (3)	
H17	1.1144	0.7771	1.1204	0.148*	
C18	1.0230 (5)	0.8124 (7)	0.9395 (7)	0.112 (3)	
H18	0.9739	0.7586	0.9472	0.134*	
N1	0.4441 (3)	0.8336 (3)	−0.0228 (3)	0.0349 (9)	
N2	0.5866 (3)	0.9241 (3)	0.1689 (4)	0.0390 (9)	
N3	0.6254 (3)	0.7724 (3)	0.2965 (3)	0.0336 (9)	
N4	0.6623 (3)	0.9535 (3)	0.2762 (4)	0.0406 (10)	
N5	1.1795 (4)	0.8996 (5)	1.0499 (5)	0.0778 (15)	
O1	0.3799 (3)	1.0467 (3)	0.1325 (4)	0.0501 (10)	
Zn1	0.5000	1.0000	0.0000	0.0435 (3)	
H1W	0.376 (5)	1.109 (5)	0.141 (6)	0.080*	
H2W	0.328 (5)	1.006 (5)	0.139 (6)	0.080*	

### Atomic displacement parameters (Å^2^)

**Table:**

	*U*^11^	*U*^22^	*U*^33^	*U*^12^	*U*^13^	*U*^23^
C1	0.037 (3)	0.039 (2)	0.035 (3)	0.004 (2)	−0.014 (2)	0.003 (2)
C2	0.051 (3)	0.040 (3)	0.035 (3)	−0.009 (2)	−0.011 (2)	−0.006 (2)
C3	0.056 (3)	0.035 (2)	0.045 (3)	−0.007 (2)	−0.007 (3)	0.002 (2)
C4	0.048 (3)	0.033 (2)	0.036 (3)	0.001 (2)	−0.008 (2)	0.005 (2)
C5	0.035 (2)	0.032 (2)	0.026 (2)	0.0006 (18)	−0.001 (2)	−0.0002 (18)
C6	0.030 (2)	0.029 (2)	0.031 (2)	0.0004 (17)	−0.001 (2)	0.0008 (18)
C7	0.036 (3)	0.036 (2)	0.032 (3)	0.0013 (19)	−0.003 (2)	−0.002 (2)
C8	0.033 (3)	0.044 (3)	0.031 (2)	−0.002 (2)	−0.005 (2)	0.000 (2)
C9	0.068 (4)	0.041 (3)	0.060 (4)	−0.009 (3)	−0.031 (3)	0.010 (3)
C10	0.065 (4)	0.053 (3)	0.058 (4)	−0.016 (3)	−0.027 (3)	0.000 (3)
C11	0.039 (3)	0.061 (3)	0.032 (3)	−0.005 (2)	−0.009 (2)	0.005 (2)
C12	0.049 (3)	0.064 (3)	0.041 (3)	−0.006 (3)	−0.011 (3)	0.019 (3)
C13	0.046 (3)	0.048 (3)	0.040 (3)	−0.011 (2)	−0.008 (2)	0.009 (2)
C14	0.038 (3)	0.074 (4)	0.039 (3)	−0.009 (3)	−0.007 (3)	0.005 (3)
C15	0.076 (5)	0.097 (5)	0.081 (5)	−0.032 (4)	−0.033 (4)	0.014 (4)
C16	0.073 (5)	0.102 (5)	0.083 (5)	−0.026 (4)	−0.026 (4)	0.006 (4)
C17	0.092 (6)	0.197 (9)	0.059 (4)	−0.042 (6)	−0.039 (4)	0.053 (5)
C18	0.070 (5)	0.191 (8)	0.058 (4)	−0.042 (5)	−0.030 (4)	0.049 (5)
N1	0.039 (2)	0.0279 (18)	0.032 (2)	0.0037 (16)	−0.0103 (17)	0.0011 (15)
N2	0.041 (2)	0.034 (2)	0.034 (2)	−0.0006 (17)	−0.0135 (18)	−0.0007 (16)
N3	0.033 (2)	0.0334 (19)	0.029 (2)	0.0020 (15)	−0.0094 (17)	0.0007 (16)
N4	0.041 (2)	0.0330 (19)	0.038 (2)	0.0003 (17)	−0.0174 (19)	0.0022 (17)
N5	0.047 (3)	0.131 (5)	0.047 (3)	−0.003 (3)	−0.015 (2)	−0.003 (3)
O1	0.052 (2)	0.0376 (18)	0.054 (2)	−0.0033 (17)	−0.0082 (18)	−0.0060 (18)
Zn1	0.0506 (5)	0.0289 (4)	0.0399 (5)	−0.0042 (4)	−0.0204 (4)	0.0043 (3)

### Geometric parameters (Å, º)

**Table:**

C1—N1	1.338 (5)	C11—C14	1.475 (6)
C1—C2	1.361 (6)	C12—C13	1.375 (6)
C1—H1	0.9300	C12—H12	0.9300
C2—C3	1.366 (6)	C13—H13	0.9300
C2—H2	0.9300	C14—C18	1.341 (7)
C3—C4	1.385 (6)	C14—C15	1.368 (8)
C3—H3	0.9300	C15—C16	1.389 (8)
C4—C5	1.370 (6)	C15—H15	0.9300
C4—H4	0.9300	C16—N5	1.306 (8)
C5—N1	1.348 (5)	C16—H16	0.9300
C5—C6	1.477 (5)	C17—N5	1.289 (8)
C6—N2	1.327 (5)	C17—C18	1.397 (8)
C6—N3	1.338 (5)	C17—H17	0.9300
C7—N4	1.335 (5)	C18—H18	0.9300
C7—N3	1.361 (5)	N1—Zn1	2.134 (3)
C7—C8	1.469 (6)	N2—N4	1.358 (5)
C8—C13	1.378 (6)	N2—Zn1	2.048 (3)
C8—C9	1.381 (6)	O1—Zn1	2.301 (4)
C9—C10	1.374 (7)	O1—H1W	0.75 (6)
C9—H9	0.9300	O1—H2W	0.85 (6)
C10—C11	1.384 (7)	Zn1—N2^i^	2.048 (3)
C10—H10	0.9300	Zn1—N1^i^	2.134 (3)
C11—C12	1.376 (6)	Zn1—O1^i^	2.301 (4)
			
N1—C1—C2	122.7 (4)	C15—C14—C11	120.6 (5)
N1—C1—H1	118.6	C16—C15—C14	120.5 (6)
C2—C1—H1	118.6	C16—C15—H15	119.8
C3—C2—C1	118.8 (4)	C14—C15—H15	119.8
C3—C2—H2	120.6	N5—C16—C15	124.9 (7)
C1—C2—H2	120.6	N5—C16—H16	117.6
C2—C3—C4	119.9 (4)	C15—C16—H16	117.6
C2—C3—H3	120.1	N5—C17—C18	125.8 (7)
C4—C3—H3	120.1	N5—C17—H17	117.1
C5—C4—C3	118.2 (4)	C18—C17—H17	117.1
C5—C4—H4	120.9	C14—C18—C17	120.3 (7)
C3—C4—H4	120.9	C14—C18—H18	119.9
N1—C5—C4	122.2 (4)	C17—C18—H18	119.8
N1—C5—C6	113.4 (3)	C1—N1—C5	118.2 (4)
C4—C5—C6	124.4 (4)	C1—N1—Zn1	127.1 (3)
N2—C6—N3	112.9 (3)	C5—N1—Zn1	114.6 (3)
N2—C6—C5	118.1 (3)	C6—N2—N4	107.3 (3)
N3—C6—C5	128.9 (4)	C6—N2—Zn1	115.6 (3)
N4—C7—N3	113.3 (4)	N4—N2—Zn1	137.1 (3)
N4—C7—C8	119.9 (4)	C6—N3—C7	101.7 (3)
N3—C7—C8	126.7 (4)	C7—N4—N2	104.8 (3)
C13—C8—C9	117.6 (4)	C17—N5—C16	113.3 (6)
C13—C8—C7	123.5 (4)	Zn1—O1—H1W	112 (5)
C9—C8—C7	119.0 (4)	Zn1—O1—H2W	123 (4)
C10—C9—C8	121.5 (5)	H1W—O1—H2W	119 (6)
C10—C9—H9	119.3	N2^i^—Zn1—N2	180.0
C8—C9—H9	119.3	N2^i^—Zn1—N1	101.80 (13)
C9—C10—C11	121.6 (5)	N2—Zn1—N1	78.20 (13)
C9—C10—H10	119.2	N2^i^—Zn1—N1^i^	78.20 (13)
C11—C10—H10	119.2	N2—Zn1—N1^i^	101.80 (13)
C12—C11—C10	116.1 (4)	N1—Zn1—N1^i^	180.000 (1)
C12—C11—C14	124.0 (4)	N2^i^—Zn1—O1^i^	89.48 (14)
C10—C11—C14	119.9 (5)	N2—Zn1—O1^i^	90.52 (14)
C13—C12—C11	123.1 (4)	N1—Zn1—O1^i^	88.42 (13)
C13—C12—H12	118.5	N1^i^—Zn1—O1^i^	91.58 (13)
C11—C12—H12	118.5	N2^i^—Zn1—O1	90.52 (14)
C12—C13—C8	120.2 (4)	N2—Zn1—O1	89.48 (14)
C12—C13—H13	119.9	N1—Zn1—O1	91.58 (13)
C8—C13—H13	119.9	N1^i^—Zn1—O1	88.42 (13)
C18—C14—C15	114.4 (5)	O1^i^—Zn1—O1	180.000 (1)
C18—C14—C11	124.8 (5)		

Symmetry code: (i) −*x*+1, −*y*+2, −*z*.

### Hydrogen-bond geometry (Å, º)

**Table:**

*D*—H···*A*	*D*—H	H···*A*	*D*···*A*	*D*—H···*A*
O1—H1*W*···N3^ii^	0.75 (6)	2.07 (6)	2.812 (5)	169 (6)
O1—H2*W*···N5^iii^	0.85 (6)	2.38 (6)	3.165 (7)	155 (6)

Symmetry codes: (ii) −*x*+1, *y*+1/2, −*z*+1/2; (iii) *x*−1, *y*, *z*−1.
